# Supplementary material for: Epidemiology of infection by pulmonary non-tuberculous mycobacteria in French Guiana 2008–2018
Source: PLoS Negl Trop Dis. 2022 Sep 9;16(9):e0010693. doi: 10.1371/journal.pntd.0010693 (PMC9491559; doi:10.1371/journal.pntd.0010693)
Supplement: Attachment — Submitted filename: 2_revision_PLOS_list of responses for reviewers.docx [file pntd.0010693.s001.docx]

Dear PLOS Neglected Tropical Disease reviewers,

Thank you for your comments.

Detailed list of responses to the review comments:

<Summary>

“was the most frequently isolated pathogen (38%)”: italics removed

“The incidence of NTM-PD is as much or even higher than metropolitan France” changed by “Although French Guiana is the French territory with the highest tuberculosis incidence, NTM should not be overlooked since their incidence is comparable to other countries and metropolitan France.”

*Arguments:* effectively, incidence rate seems to be similar when compared to same period

<Introduction>

Add: “Buruli ulcer, caused by *Mycobacterium ulcerans*, is well known in French Guiana to lead tissue destruction, resulting in skin ulcers and sometimes bone infection (2).”

“French Guiana is the region of France with the highest Tuberculosis incidence (32.5 per 100,000 inhabitants”.

Add precision: “in 2017”

<Methods>

*Reviewer #3:* “The authors state that this their studies were notified to the "National Commission for Data Protection", but it is unknown if this study was approved by a governing board (such as an institutional review board or IRB) for human protections or if there was any study protocols which took safeguards into account for this project. There is no indication that there are ethical issues, but this should be explained in the Methods section of the manuscript.”

*Response:* declaration number and date of approvment specified page 5: “The main investigators don’t have any ethical problem in the study. It was declared to the National Commission for Data Protection (CNIL) and was approved on April 19, 2019 (declaration number: 2212828v0).”

Major letter added for Chi2 and Fisher test

<Results>

*Reviewer #3*: “Tables were adequate, however there should be legends for both the tables and figures. For the figures: Figure 1 appeared to be of poor resolution and thus was blurry to read; Figure 2 had line markers that were difficult to see, and thus using a different scheme for the appearance of the two lines would be better; Figure 3 was satisfactory, yet the font color for the numbers within the pie charts were difficult to read against the background and thus should be more uniform with a darker color.”

*Responses*: Figure 1, 2 and 3 modified.

*Figure 3:* the total number of isolates is 177 because the place of residence was unknown for one of the patients.

<Conclusion>

*Reviewer #2:* “The impact on public health is not discussed in detail and the authors do not really discuss how the results of their study advances what is known about NTMs. However, the study is the first to investigate and report on the prevalence of NTMs in French Guiana, it does have significant public health issues for the region.”

*Responses*: Geographical, environmental and clinical differences between French Guiana and metropolitan France were supported and explained microbiological differences.

*Reviewer #3:* “A good example of this is noted on Pages 8-10. The authors go from documenting Canadian rates of NTM prevalence and then diving into France  and what is seen there to coastal Brazil and then back to France when it comes to their MTB prevalence. Also there is no data discussed about what is transpiring in other nations such as the United States, Mexico or other countries of South America. Furthermore, there are minimal mentioning of association with other diseases which are more frequently associated with NTM mentioned in the manuscript (as was illustrated in Table 1) »

*Responses*: Presentation of discussion have been reorganized. Firstly, comparison French Guiana incidence to those in metropolitan France and other countries. Secondly, explanation of progressive increasing incidence and possible causes of lower incidence than in mainland France. Finally, explanation of microbiological differences between France and neighbours because of geographical, environmental and clinical differences.

Reviewer #3: “Also there is no data discussed about what is transpiring in other nations such as the United States, Mexico or other countries of South America. Furthermore, there are minimal mentioning of association with other diseases which are more frequently associated”

*Response:* I add: “In Argentina, the most important pathogen was *MAC* particularly because of HIV co-infected patients (32). In Colombia, *MAC* and *M. abscessus* were predominant too: a history of tuberculosis and HIV infection were describe to promote disease (33). 16% of patients of this study have a history of tuberculosis and 46% have HIV infection”.

*Reviewer #3:* “One would ask the authors if there was any data to suggest that M. xenopi is seen in more temperate/cooler climates compared to M. abscessus which is seen in more tropical/subtropical climates? Data from Rebecca Prevots group at National Institutes of Health (USA) and Theodore Marras' group at University of Toronto have both looked at this in the past. Both are in the references, but their data is important to review and thus make the argument for the rigor of what is documented in these data.”

*Response*: I added such reference about it:

- “Some studies reported it in the USA where NTM-PD were more predominant on the coast and Hawaii (36,37). As explained by Prevots and al in the USA, using municipal water supply could also explain PNTM were more isolated on the coastal center area in French Guiana, where the main city Cayenne is situated.

- “*M. abscessus* was only found on the coastal center region in French Guiana. Adjemian and al showed a highest prevalence of *M. abscessus* around the tropical climate”

*Reviewer #3:* “Finally, would include any supporting conclusions about whether soil composition, potable water methods used in French Guiana or other factors could be supporting the results seen in this investigation. This is touched upon, but there is no real hypothesis and literature review regarding this topic. Manuscripts by Joseph Falkinham's group has documented this well and has reasoning for potential hypotheses that would make the case stronger for these data.”

*Response: “*Moreover, French Guiana’s soil rich in manganese, clay, and acidic pH presents coastal swampy soils (38,39). It was already described by Joseph Falkinham’s works”

<Editorial and data presentation modifications>

Reviewer #2 and Responses:

1) 1.1.1.1.1 in the CCI score removed.

2) Missing one stroke: added

3) PNTM chosen

4) Number of patients in each category suppressed

5) Results table 3 detailed

6) Bold suppressed

7) modified

8) One patient missing data about it

<b>Summary and General Comments</b>

Reviewer #1: “the introduction should explicitly mention that the particular amazonian ecosystem makes the question interesting at least in my view this is what the added value of the paper is”

*Response*: Addition “There are some environmental and socio-demographical differences with mainland France”

Reviewer #1: “the authors mention that "This could be one of the explanations of the

low proportion of M. kansasii found in French Guiana since this species is the adage of HIVpositive patients with CD4> 200 / mm3 (18). " i think this is wrong because most hiv patients in french guiana have cd4 counts above 200

*Response*: I suppressed it and proposed another hypothesis.

Reviewer #2: “For example, on page 7, the abbreviation INSEE is used. Please define this abbreviation the first time it is used.”

*Response:* INSEE was defined page 6 -7. COPD, RGM defined too.

Reviewer #2: “2) On page 10, the authors talk about a decrease in the number of HIV positive cases and says that it remains high with 743 million inhabitants (versus 89 million in mainland France). Do the authors mean that there were 743 million people wit HIV in French Guiana vs 89 Million in mainland France? This cannot be possible. Please rephrase the sentence so that it explains these numbers.”

*Response: modified by “*number of HIV positive findings in 2015: 743 against 89 per million people respectively”

Reviewer #3: “Thus, the manuscript requires major revision of the following:

1) addition of an ethical statement regarding the use of patient sensitive information and how that information was reviewed and safeguarded. This is generally important when dealing with data mined from patient charts and thus patient information should not be compromised.

2) Figures and tables need to be more clear and the data illustrated in a more appropriate form. All figures/tables should come with legends explaining the data.

3) The Discussion section appeared to not be written in the same format as the rest of the manuscript and thus needs major revision and rewording to accomplish the illustration of what these data demonstrate. Someone proficient in the scientific use of English would be helpful to have on hand to proofread the manuscript. Lastly, the data here are important to document, however the hypotheses as to why these data are the way the way they are is critical to what the authors are trying to accomplish. A more intense literature review and understanding of what is happening in environment and making the correlation with the clinical human disease is paramount.”

*Responses*:

1) I made precisions: “The main investigators don’t have any ethical problem in the study. It was declared to the National Commission for Data Protection (CNIL) and was approved on April 19, 2019 (declaration number: 2212828v0)”.

2) Done

3) Hoping to have answered your request
