# Supplementary material for: Epidemiology of infection by pulmonary non-tuberculous mycobacteria in French Guiana 2008–2018
Source: PLoS Negl Trop Dis. 2022 Sep 9;16(9):e0010693. doi: 10.1371/journal.pntd.0010693 (PMC9491559; doi:10.1371/journal.pntd.0010693)
Supplement: Attachment — Submitted filename: 4-revision-PLOS-comments-R3.docx [file pntd.0010693.s003.docx]

R3 révision

Dear PLOS Neglected Tropical Disease reviewers,

Thank you for your comments.
Detailed list of responses to the review comments:

**Methods**

Reviewer #3: The methods of this revised manuscript were not really modified, but requested changes were made to the points from the reviewers. This includes a statement about the institutional review board for these types of studies that was requested by this reviewer. As mentioned previously, the study design does address the stated objectives and discusses the methods used in this epidemiological analysis.

**Response : the “ethic statement” section was modified, that way:**

***“Ethical aspects***

**“This is a retrospective, non-interventional study. Data were anonymized for all patients. We solely collected necessary data for the purpose of the research. These data are archived for 15 years. This research was in compliance with the law "Informatique et Libertés" of January 6, 1978 as amended and the law No. 2018-493 of June 20, 2018 on the protection of personal data. Is also in compliance with European Parliament guidelines in April 27, 2016. Finally, data were transferred and collected in accordance with the reference methodology MR003 of the Commission Nationale de l'Informatique et des Libertés (CNIL) for which the Centre Hospitalier de Cayenne has signed a compliance commitment. The database was declared to the CNIL and approved on April 19, 2019 (declaration number: 2212828v0).”**

**Results:**

Reviewer #3: The revisions made by authors as suggested by the reviewers were done and more. The results go along with the aims and hypotheses made by the authors at the beginning. The figures still are of low quality for PLOS NTD and would strongly advise the authors to counsel with a professional at making figures for journals for a revision. These figures are what readers of PLOS NTD are going to be looking at while reading the prose of the manuscript. Quality control for journals can only do so much when the figures are out of focus based on the medium in which they were created.

**Response: I changed my numbers (tables) and figures from Apex.PACE. Hope this fits the PLOS NTD criteria.**

**Conclusions:**

Reviewer #3: Yes, the conclusions are supported by the data analysis and confirm the authors suspicions based on the climate and the socioeconomic status of the investigation subjects. The comparisons to both it's southern neighbor and mainland France do offer some perspective on this. It would be interesting to also note some of the basic information about water temperature in French Guiana and if water heaters are used as compared to mainland France. This is the hypothesis of Dr. Marras and colleagues at U Toronto. Some basic information on this as well as how much of the country uses well water or rain catchment for their fresh water source would be helpful in solidifying the conclusions based on their hypotheses.

**Response: Your comment is very relevant. Thise information was addes in the manuscript :**

**« The temperature of the sea water in French Guiana is quite stable, generally around 27-28°C, and that of the rivers and creeks a little cooler. Because of the high temperature throughout the year, water heaters are used much less than in mainland France. In coastal towns and villages, residential areas are equipped with running water. Otherwise, in the main towns slum, rainwater is often used. In the remote communities far from the coast, rainwater collection tanks and small boreholes are used for daily water supply. »**

Reviewer #3: Please see above in the 'Conclusions' section of the review. English used in the manuscript is better, yet would still suggest that this is reviewed by one who has published in an English-language journal to optimize the vernacular and enhance the point they are trying to make with this manuscript.

**Response: this article was proofread again by a second reader in order to be able to improve the English. I hope this one better meets your expectations. On the manuscript, the changes have been highlighted.**

**Summary and General Comments**

Reviewer #3: This is the second review for the manuscript entitled, "Epidemiology of infection by pulmonary non-tuberculous mycobacteria in French Guiana 2008-2018." The manuscript was reviewed previously by this reviewer; the premise of the study is to determine the epidemiology of non-tuberculous mycobacterial disease in French Guiana, which is part of France. The aim and hypotheses are the same as was the data analysis, which demonstrates that Mycobacterium avium Complex and Mycobacterium abscessus dominate as opposed to mainland France which has more M xenopi as part of the infections of NTM there. This is an important study as it demonstrates data from a part of South America which has minimal reporting.

The manuscript still has some lingering issues; those being manuscript English, details about fresh water source for the populations in the various municipalities and Figures, which need to be improved dramatically for PLOS publication standards.

Minor revision should be instituted for the above commentary prior to acceptance.

**Response: thank you very much for your requests for minor révisions**

**Figure**

Figure 3: please (a) provide a direct link to the base layer of the map used and ensure this is also included in the figure legend; (b) provide a link to the terms of use / license information for the base layer. We cannot publish proprietary or copyrighted maps (e.g. Google Maps, Mapquest) and the terms of use for your map base layer must be compatible with our CC-BY 4.0 license.

**Response : We added on legend « ** Map was created using BdCarto NGI’s (National Geographic Institute) map database”***

Note: it’s not a copyright. Our map was created by Map info for the study.

**Tables**

2) Tables should not be uploaded as individual files. Please remove these files and include the Tables in your manuscript file as editable, cell-based objects. For more information about how to format tables, see our guidelines: [https://journals.plos.org/plosntds/s/tables](https://journals.plos.org/plosntds/s/tables)

**Response : I am also sending you the manuscript with the tables but the last time I did it the format did not match and I cannot send it in tif format associated with the manuscript**

**Autors**

I preferred to add Hugo Testaert and Mathieu nacher in the acknowledgments section

**Other comments (June 4^th^ 2022):**

“Before the journal can consider a final decision of acceptance, English language editing MUST be done. The journal does not copy edit papers, leaving the final version to the reviewrs. There are problems with italicing the species names, agree of nouns and adjectives etc. Please have either a professional editor or someone with advanced English writing expertised go over the ms. »

**Response:** **this article was proofread again by a third reader Sadia Khan**
